# (*S*)-(+)-1-(4-Bromo­phen­yl)-*N*-[(4-methoxyphen­yl)methyl­idene]ethyl­amine and bis­{(*S*)-(+)-1-(4-bromo­phen­yl)-*N*-[(4-methoxyphen­yl)methyl­idene]ethyl­amine-κ*N*}di­chlorido­palladium(II)

**DOI:** 10.1107/S2056989024000690

**Published:** 2024-01-26

**Authors:** Bertin Anzaldo, Gloria E. Moreno Morales, Claudia P. Villamizar C., Ángel Mendoza, Guadalupe Hernández Téllez

**Affiliations:** a Lab. Síntesis de Complejos, Fac. Cs. Quím.–BUAP, Ciudad Universitaria, PO Box 72592 Puebla, Mexico; b Instituto de Química Universidad Autónoma de México UNAM, Circuito Exterior Cd Universitaria, PO Box 04510, Ciudad de México, Mexico; Universidade Federal do ABC, Brazil

**Keywords:** crystal structure, Schiff base, palladium(II) com­plex, monodentate

## Abstract

The (*S*)-(+)-1-(4-bromo­phen­yl)-*N*-[(4-methoxyphen­yl)methyl­idene]ethyl­amine ligand, C_16_H_16_BrNO, (I), was synthesized through the reaction of 4-meth­oxy­anisaldehyde with (*S*)-(−)-1-(4-bromo­phen­yl)ethyl­amine. It crystallizes in the ortho­rhom­bic space group *P*2_1_2_1_2_1_ belonging to the Sohncke group, featuring a single mol­ecule in the asymmetric unit. The refinement converged successfully, achieving an *R* factor of 0.0508. The Pd^II^ com­plex bis­{(*S*)-(+)-1-(4-bromo­phen­yl)-*N*-[(4-methoxyphen­yl)methyl­idene]ethyl­amine-κ*N*}di­chlorido­pal­ladium(II), [PdCl_2_(C_16_H_16_BrNO)_2_], (II), crystallizes in the monoclinic space group *P*2_1_ belonging to the Sohncke group, with two mol­ecules in the asymmetric unit. The central atom is tetra­coordinated by two N atoms and two Cl atoms, resulting in a square-planar configuration. The imine moieties exhibit a *trans* configuration around the Pd^II^ centre, with average Cl—Pd—N angles of approximately 89.95 and 90°. The average distances within the palladium com­plex for the two mol­ecules are ∼2.031 Å for Pd—N and ∼2.309 Å for Pd—Cl.

## Abstract 2

The synthesis, crystal structure and analysis of a chiral Schiff base (*S*)-(+)-1-(4-bromo­phen­yl)-*N*-[(4-methoxyphen­yl)methyl­idene]ethyl­amine ligand along with its corresponding palladium(II) com­plex are detailed. The crystal structures exhibit monoclinic *P*2_1_ and ortho­rhom­bic *P*2_1_2_1_2_1_ symmetries, respectively. The structure of the palladium(II) com­plex reveals C—H⋯O and C—H⋯Br hydrogen-bonding inter­actions involving two distinct mol­ecules within the asymmetric unit.

## 1. Chemical context

Schiff base ligands commonly result from the condensation of primary amines and aldehydes. The ease of their synthesis and the flexibility of their chemical structures make Schiff bases widely used in coordination chemistry, with a wide range of coordination com­plexes (Boulechfar *et al.*, 2023 ▸). The catalytic prowess of Schiff base com­plexes with metal centres is well documented and shows enhanced activity in various chemical reactions (Gupta & Sutar, 2008 ▸). Their catalytic potential extends to processes such as oxidation, hy­droxy­lation, aldol condensation and epoxidation (Brayton *et al.*, 2009 ▸; Hu *et al.*, 2016 ▸; Bowes *et al.*, 2011 ▸). Changes in the substituents of the imine com­pounds affect their reactivity, influenced by electronic and steric factors that affect their structure. In particular, some imine com­pounds present conjugated electron systems and have attracted attention for their optical and materials properties (Kalita *et al.*, 2014 ▸; Anzaldo *et al.*, 2019 ▸; Cîrcu *et al.*, 2006 ▸). The presence of chirality in the structures enhances a valuable dimension for catalyst design, allowing for fine-tuning and selectivity in a variety of chemical reactions. Here we report the crystal and mol­ecular structure of the chiral Schiff base (*S*)-(+)-1-(4-bromo­phen­yl)-*N*-[(4-methoxyphen­yl)methyl­idene]ethyl­amine, (I), and its palladium(II) com­plex, bis­{(*S*)-(+)-1-(4-bromo­phen­yl)-*N*-[(4-methoxyphen­yl)methyl­idene]ethyl­amine-κ*N*}di­chlorido­pal­la­dium(II), (II), which has not been reported previously. 
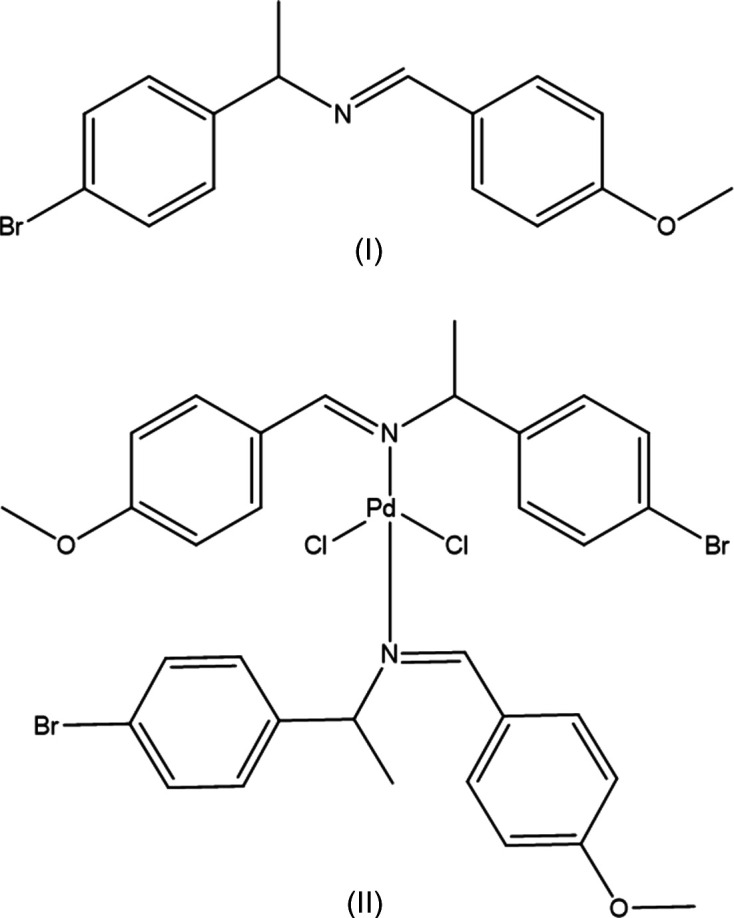


## 2. Structural commentary

**Figure 1:**
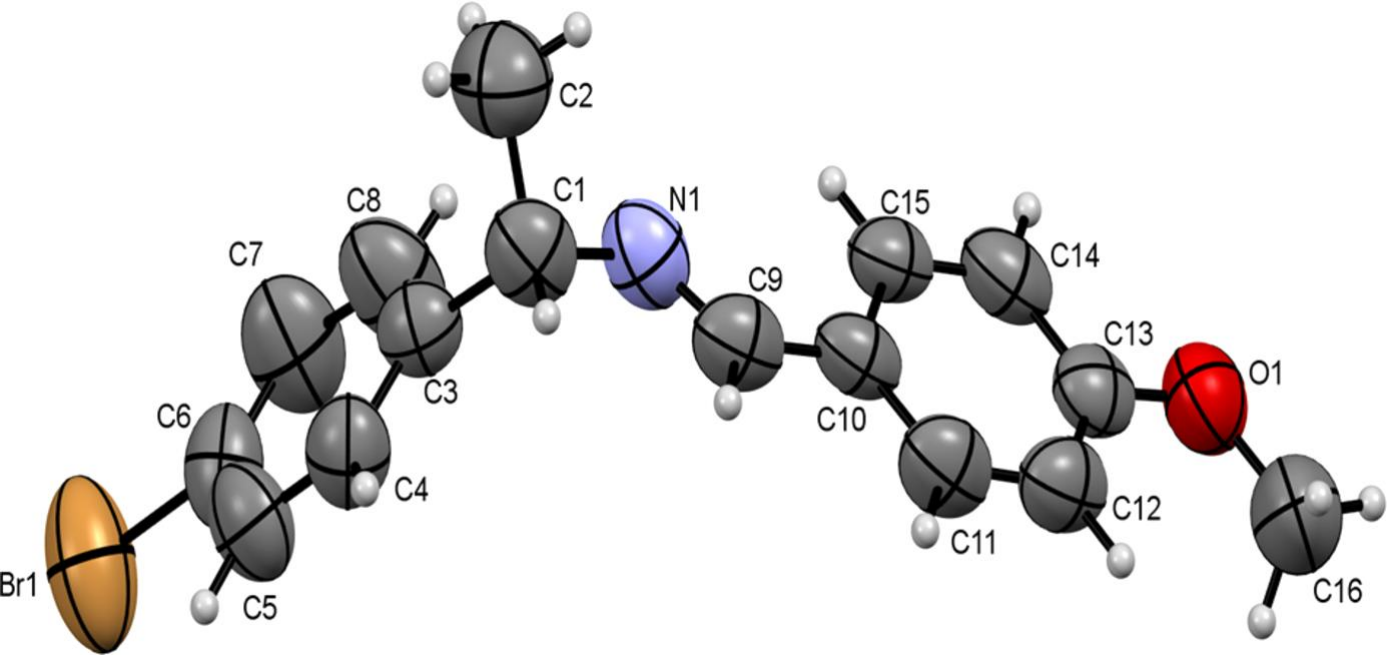
The mol­ecular structure of (*S*)-(+)-1-(4-bromo­phen­yl)-*N*-[(4-methoxyphen­yl)methyl­idene]ethyl­amine ligand, (I). Displacement ellipsoids are drawn at the 50% probability level.

The ligand crystallizes in the ortho­rhom­bic system with the space group *P*2_1_2_1_2_1_. Within the asymmetric unit, there is a single mol­ecule, as depicted in Fig. 1 ▸. The length of the C9=N1 double bond is 1.265 (7) Å. The imine group exhibits a C1—N1—C9 angle of 118.1 (6)°. The bond lengths and angles confirm the *sp* ^2^ hybridization for the C and N atoms.

**Figure 2:**
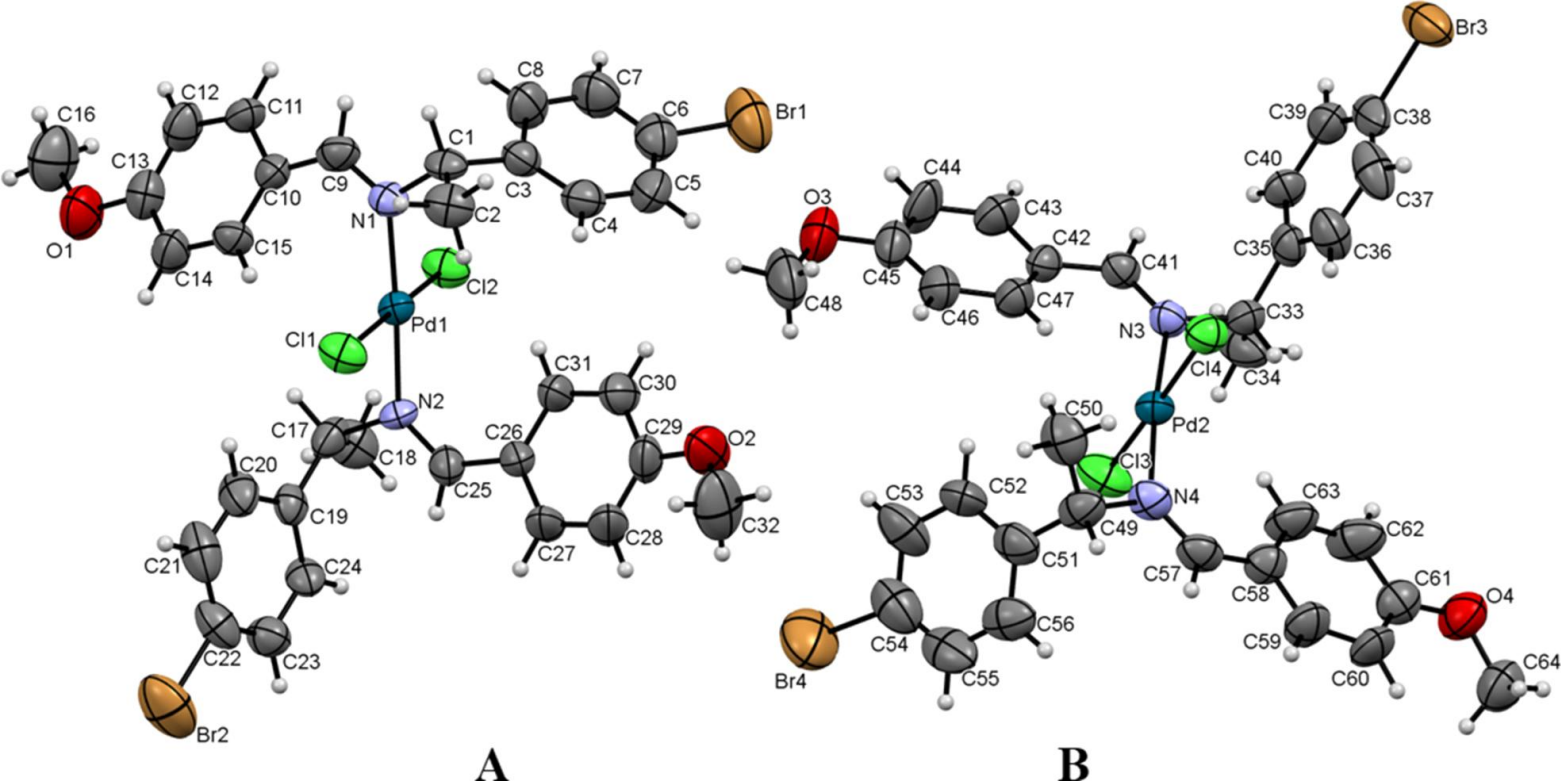
The mol­ecular structure of the two mol­ecules units in the asymmetric unit of the title palladium(II) com­plex, (II). Displacement ellipsoids are drawn at the 50% probability level.

The palladium(II) com­plex crystallizes within the monoclinic system, space group *P*2_1_. The structure contains two independent mol­ecules (labelled as *A* and *B*) within the asymmetric unit, as illustrated in Fig. 2 ▸. The length of the C=N bond is com­parable to that observed in the ligand.

The steric effects induced by coordination in the Pd^II^ com­plex are evident in the torsion angles for mol­ecule *A* of C15—C10—C9—N1 = 29.4 (16)° and C31—C26—C25—N2 = −23.0 (16)°, and for mol­ecule B of C47—C42—C41—N3 = 15.9 (16)° and C63—C58—C57—N4 = −3(2)°, as com­pared with the ligand C15—C10—C9—N1 torsion angle of 7.2 (9)°. The average bond angle within the imine group is 117.03°, and the average bond distance at the imine group (C=N) is 1.285 Å. These bond lengths and angles provide confirmation of the *sp* ^2^ hybridization of the C and N atoms. The crystal structure of the Pd^II^ com­plex shows disorder in the two Br atoms in mol­ecule *B* of the asymmetric unit.

## 3. Supra­molecular features

**Figure 3:**
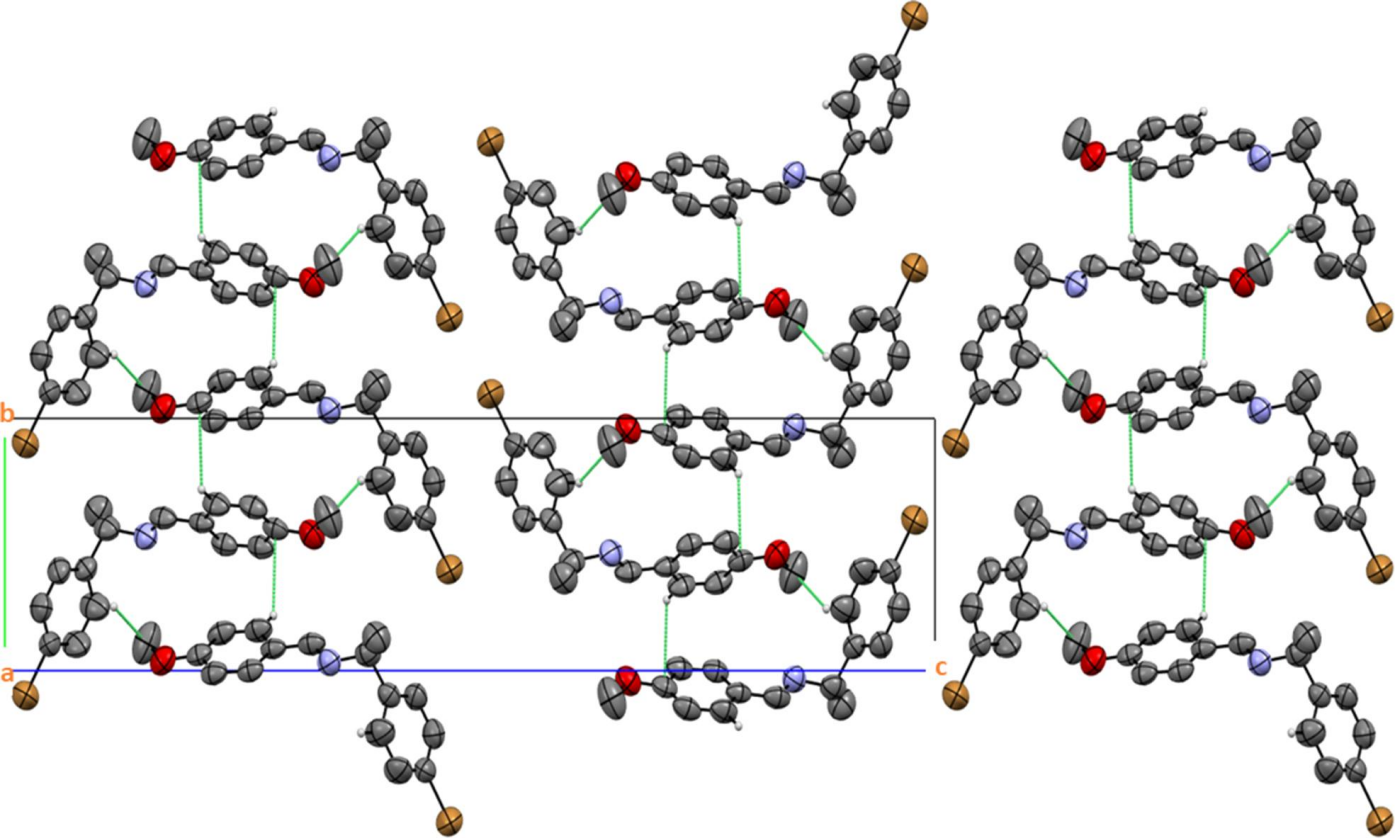
Growth in the projection on the *bc* plane (displacement ellipsoids are presented with 50% probability), with dashed lines indicating inter­molecular contacts. All H atoms not involved in these inter­actions have been omitted for clarity.

The arrangement of the ligand mol­ecule arises from short contacts corresponding to van der Waals inter­actions. Inter­molecular distances are calculated from atomic coordinate translations along the *a* axis, revealing short C—H⋯C contacts (Nishio 2004 ▸; Enamullah *et al.*, 2007 ▸; Brandl *et al.*, 2001 ▸). Specific inter­actions include H11⋯C13 at 2.855 Å and H8⋯C16 at 2.836 Å, as shown in Fig. 3 ▸.

**Figure 4:**
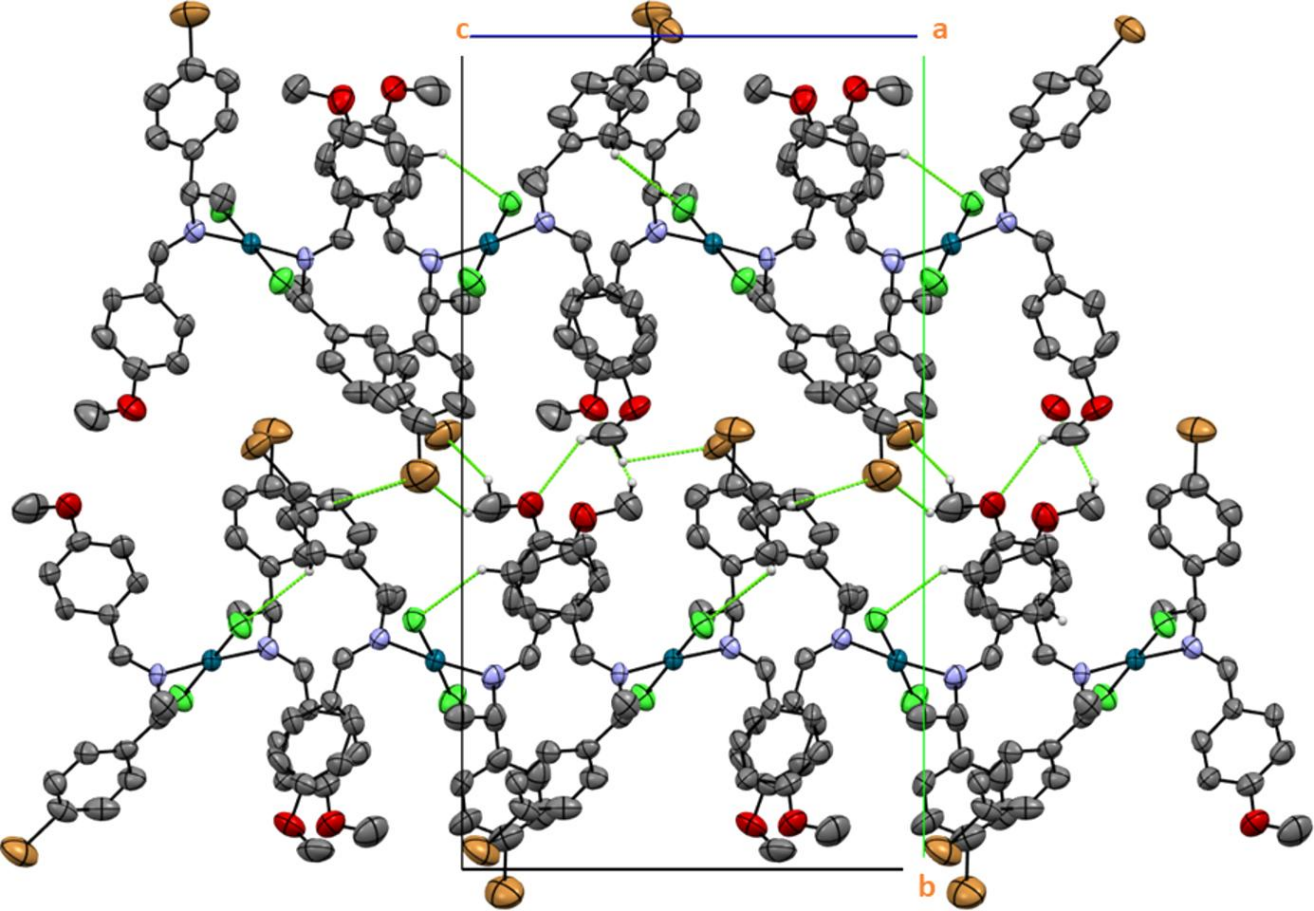
The crystal packing diagram of palladium(II) com­plex (II). The dashed lines indicate inter­molecular hydrogen bonds (displacement ellipsoids are presented with 50% probability). All H atoms not involved in these inter­actions have been omitted for clarity.

The self-assembly of the palladium(II) com­plex forms a three-dimensional structure through inter­molecular hydrogen bonds involving C—H⋯O, C—H⋯Cl, C—H⋯Br and C—H⋯C inter­actions (Desiraju, 1996 ▸; Steiner, 1997 ▸; Kinzhalov *et al.*, 2019 ▸). As a result, a packing arrangement of supra­molecular layers is produced, as depicted in Fig. 4 ▸. The mol­ecular array is influenced by all the contacts, as detailed in Table 1 ▸.

## 4. Database survey

A search of the Cambridge Structural Database (CSD, Version 5.42, current as of November 2023; Groom *et al.*, 2016 ▸) yielded five entries related to ligand (I). BUWBIG (Khalaji *et al.*, 2015 ▸), EDORUL (Enamullah *et al.*, 2007 ▸), QEQZUI (Xu *et al.*, 2006 ▸), UJUFEM (Hernández-Téllez *et al.*, 2016 ▸) and QEVTIV (Chatziefthimiou *et al.*, 2006 ▸). In the crystal structure of BUWBIG (*P*2_1_), the three-dimensional packing is stabilized by inter­molecular hydrogen bonding of the O—H⋯N and C—H⋯O types. EDORUL (*P*2_1_2_1_2_1_) exhibits influence from a C—H⋯π inter­action, with a C—H⋯π plane angle of 52°, as well as C—Br⋯π contacts to the salicyl ring, with a C—Br⋯centroid angle of 166.0° and a C—Br⋯π angle of 73.4°. The asymmetric unit of QEQZUI (*Pbca*) com­prises one mol­ecule in an ortho­rhom­bic crystal system. In UJUFEM (*P*2_1_2_1_2_1_), the chiral C atom is in the *R* configuration, and the benzene ring is *para*-substituted by a methoxy group. QEVTIV (*P*2_1_2_1_2_1_) mol­ecules are stabilized by inter­molecular hydrogen bonding of the O—H⋯N and C—H⋯O types. Crystal structures for chiral imines derived from 4-meth­oxy­anisaldehyde are relatively scarce com­pared to the extensive chemistry of Schiff bases.

In the case of the com­plex of Pd^II^, some previously reported structures include LATNAV (Rochon *et al.*, 1993 ▸), in which the structure is stabilized through hydrogen-bonding inter­actions between the hy­droxy groups and the chloride ligands, with the Pd^II^ ion exhibiting square-planar coordination geometry around the metal centre in the space group *P*2_1_/*c*. FATQAU and FATPUN (Motswainyana *et al.*, 2012*b* ▸) crystallizes in the space group *P*2_1_/*n*. The two mol­ecular structures exhibit square-planar geometry around the Pd atom. In each mol­ecule, the Pd atom is coordinated to two *trans*-ferrocenyl­imine mol­ecules *via* their imine N atoms, and either two chlorides or a chloride and a methyl. UQUFIW (Duong *et al.*, 2011 ▸) crystallizes in the space group *P*1. The chloride and (pyridin-4-yl)boronic acid ligands adopt a *trans* arrangement due to mol­ecular symmetry, and angles are about 90°. YATQAN (Motswainyana *et al.*, 2012*a* ▸) crystallizes in the space group *P*2_1_/*n*. The Pd^II^ ion has square-planar coordination geometry around the metal centre, coordinated to two ferrocenyl­imine ligands *via* the imine N atoms and the chloride ions. The ferrocenyl­imine mol­ecules are *trans* with respect to each other across the centre of symmetry.

## 5. Synthesis and crystallization

Under solvent-free conditions, a mixture of (*S*)-(–)-1-(4-bromo­phen­yl)ethyl­amine (0.279 g, 1.39 mmol) and 4-meth­oxy­anisaldehyde (0.190 g, 1.39 mmol) in a 1:1 molar ratio were mixed at room temperature, giving a white solid. The crude was recrystallized from CH_2_Cl_2_ by slow evaporation, affording colourless crystals of the ligand (I) (yield 93%; m.p. 51–53 °C).

FT–IR (cm^−1^): 1644 cm^−1^ (C=N); ^1^H NMR (500 MHz, CDCl_3_/TMS): δ 8.28 (*s*, 1H; *H*C=N), 7.73–7.70 (*m*, 2H; Ar-*H*), 7.46–7.43 (*m*, 2H; Ar-*H*), 7.32–7.29 (*m*, 2H; Ar-*H*), 6.93–6.90 (*m*, 2H; Ar-*H*), 4.45 (*q*, 1H; C*H*CH_3_), 3.84 (*s*, 3H; OC*H*_3_), 1.535 (*d*, 3H; C*H*_3_); ^13^C NMR (500 MHz, CDCl_3_/TMS): δ 161.69 (H*C*=N), 159.12, 144.53, 131.44, 129.85, 129.19, 128.42, 120.47, 113.97 (*C*-Ar), 68.98 (*C*HCH_3_), 55.39 (*OC*H_*3*_), 24.97 (CH*C*H_3_) ppm. (ESI^+^): *m*/*z* calculated for C_16_H_16_BrNO: 318.2140 found 318. [α]^20^_D_ = +80.13 (*c* = 1, CHCl_3_).

To a solution of bis­(benzo­nitrile)­palladium(II) chloride (0.050 g, 0.13 mmol) in CH_2_Cl_2_ (5 ml) was added a solution of (*S*)-(+)-[1-(4-bromo­phenyl)-*N*-(4-meth­oxy­phen­yl)methyl­idene]ethyl­amine (0.082 g, 0.26 mmol) in CH_2_Cl_2_ (10 ml). The solution was stirred for 12 h to give a light-orange precipitate. The precipitate was filtered off to obtain a light-orange solid. Recrystallization from a mixture of CH_2_Cl_2_ and hexane afforded single crystals suitable for X-ray analysis.

## 6. Refinement

Crystal data, data collection and structure refinement details are summarized in Table 2 ▸. H atoms were positioned geometrically and refined as riding [C—H = 0.93–0.93 Å with *U*_iso_(H) = 1.2*U*_eq_(C)].

## Supplementary Material

Crystal structure: contains datablock(s) I, II, global. DOI: 10.1107/S2056989024000690/ee2003sup1.cif

Structure factors: contains datablock(s) I. DOI: 10.1107/S2056989024000690/ee2003Isup2.hkl

Structure factors: contains datablock(s) II. DOI: 10.1107/S2056989024000690/ee2003IIsup3.hkl

CCDC references: 2293931, 2293932

Additional supporting information:  crystallographic information; 3D view; checkCIF report

## Figures and Tables

**Table 1:** Hydrogen-bond geometry (Å, °) for (II)

*D*—H⋯*A*	*D*—H	H⋯*A*	*D*⋯*A*	*D*—H⋯*A*
C2—H2*C*⋯Cl1^i^	0.96	2.84	3.354	114
C7—H7⋯Br4^i^	0.93	2.61	3.269	129
C9—H9⋯Cl4^ii^	0.93	2.96	3.883	173
C18—H18⋯Cl2^iii^	0.96	2.92	3.679	137
C21—H21⋯Br3^iii^	0.93	3.01	3.646	127
C28—H28⋯Cl4^iv^	0.93	2.79	3.544	139
C32—H32*B*⋯Br2^v^	0.96	2.98	3.811	146
C32—H32*C*⋯Br4^v^	0.96	2.79	3.607	143
C34—H34*A*⋯Cl3^v^	0.96	2.94	3.709	138
C48—H48*A*⋯Br3^ii^	0.96	3.04	3.483	110
C48—H48⋯O2^v^	0.96	2.63	3.426	141
C50—H50*A*⋯Cl4^v^	0.96	2.90	3.376	112
C64—H64*A*⋯O1^vi^	0.96	2.57	3.284	131

Symmetry codes: (i) 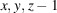 ; (ii) 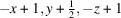 ; (iii) 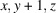 ; (iv) 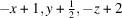 ; (v) 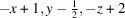 ; (vi) 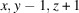 .

**Table 2:** Experimental details For all structures: *Z* = 4. Experiments were carried out at 293 K with Mo *K*α radiation using a Rigaku Xcalibur Atlas Gemini diffractometer. The absorption correction was analytical (*CrysAlis PRO*; Rigaku OD, 2015 ▸). H-atom parameters were constrained.

	(I)	(II)
Crystal data		
Chemical formula	C_16_H_16_BrNO	[PdCl_2_(C_16_H_16_BrNO)_2_]
*M*_r_	318.21	813.71
Crystal system, space group	Orthorhombic, *P*2_1_2_1_2_1_	Monoclinic, *P*2_1_
*a*, *b*, *c* (Å)	5.6599 (11), 7.9243 (10), 34.353 (5)	9.0493 (4), 25.1365 (8), 14.1725 (7)
α, β, γ (°)	90, 90, 90	90, 90.185 (4), 90
*V* (Å^3^)	1540.8 (4)	3223.8 (2)
μ (mm^−1^)	2.66	3.25
Crystal size (mm)	0.4 × 0.25 × 0.08	0.58 × 0.14 × 0.11

Data collection		
*T*_min_, *T*_max_	0.840, 0.953	0.406, 0.755
No. of measured, independent and observed [*I* > 2σ(*I*)] reflections	9633, 2852, 1534	21980, 12072, 8864
*R*_int_	0.050	0.047
(sin θ/λ)_max_ (Å^−1^)	0.607	0.625

Refinement		
*R*[*F* ^2^ > 2σ(*F* ^2^)], *wR*(*F* ^2^), *S*	0.051, 0.116, 1.05	0.047, 0.107, 1.02
No. of reflections	2852	12072
No. of parameters	175	767
No. of restraints	0	41
Δρ_max_, Δρ_min_ (e Å^−3^)	0.21, −0.21	0.78, −0.64
Absolute structure	Flack *x* determined using 403 quotients [(*I* ^+^)−(*I* ^−^)]/[(*I* ^+^)+(*I* ^−^)] (Parsons *et al.*, 2013 ▸)	Flack *x* determined using 2945 quotients [(*I* ^+^)−(*I* ^−^)]/[(*I* ^+^)+(*I* ^−^)] (Parsons *et al.*, 2013 ▸)
Absolute structure parameter	−0.026 (9)	0.005 (7)

Computer programs: *CrysAlis PRO* (Rigaku OD, 2015 ▸), *SHELXS* (Sheldrick, 2008 ▸), *SHELXT* (Sheldrick, 2015*a* ▸), *SHELXL2019* (Sheldrick, 2015*b* ▸) and *OLEX2* (Dolomanov *et al.*, 2009 ▸).

## supplementary crystallographic information

### (S)-(+)-1-(4-Bromophenyl)-N-[(4-methoxyphenyl)methylidene]ethylamine (I) Crystal data

**Table:**

C_16_H_16_BrNO	*D*_x_ = 1.372 Mg m^−^^3^
*M**_r_* = 318.21	Mo *K*α radiation, λ = 0.71073 Å
Orthorhombic, *P*2_1_2_1_2_1_	Cell parameters from 2110 reflections
*a* = 5.6599 (11) Å	θ = 3.5–19.9°
*b* = 7.9243 (10) Å	µ = 2.66 mm^−^^1^
*c* = 34.353 (5) Å	*T* = 293 K
*V* = 1540.8 (4) Å^3^	Plate, clear colourless
*Z* = 4	0.4 × 0.25 × 0.08 mm
*F*(000) = 648	

### (S)-(+)-1-(4-Bromophenyl)-N-[(4-methoxyphenyl)methylidene]ethylamine (I) Data collection

**Table:**

Rigaku Xcalibur Atlas Gemini diffractometer	1534 reflections with *I* > 2σ(*I*)
Detector resolution: 5.2782 pixels mm^-1^	*R*_int_ = 0.050
ω scans	θ_max_ = 25.5°, θ_min_ = 3.1°
Absorption correction: analytical (CrysAlis PRO; Rigaku OD, 2015)	*h* = −6→6
*T*_min_ = 0.840, *T*_max_ = 0.953	*k* = −9→9
9633 measured reflections	*l* = −41→41
2852 independent reflections	

### (S)-(+)-1-(4-Bromophenyl)-N-[(4-methoxyphenyl)methylidene]ethylamine (I) Refinement

**Table:**

Refinement on *F*^2^	H-atom parameters constrained
Least-squares matrix: full	*w* = 1/[σ^2^(*F*_o_^2^) + (0.0386*P*)^2^ + 0.0945*P*] where *P* = (*F*_o_^2^ + 2*F*_c_^2^)/3
*R*[*F*^2^ > 2σ(*F*^2^)] = 0.051	(Δ/σ)_max_ < 0.001
*wR*(*F*^2^) = 0.116	Δρ_max_ = 0.21 e Å^−^^3^
*S* = 1.05	Δρ_min_ = −0.21 e Å^−^^3^
2852 reflections	Extinction correction: SHELXL2019 (Sheldrick, 2015*a*), Fc^*^=kFc[1+0.001xFc^2^λ^3^/sin(2θ)]^-1/4^
175 parameters	Extinction coefficient: 0.0040 (14)
0 restraints	Absolute structure: Flack *x* determined using 403 quotients [(I+)-(I-)]/[(I+)+(I-)] (Parsons *et al.*, 2013)
Hydrogen site location: inferred from neighbouring sites	Absolute structure parameter: −0.026 (9)

### (S)-(+)-1-(4-Bromophenyl)-N-[(4-methoxyphenyl)methylidene]ethylamine (I) Special details

**Table:**

Geometry. All esds (except the esd in the dihedral angle between two l.s. planes) are estimated using the full covariance matrix. The cell esds are taken into account individually in the estimation of esds in distances, angles and torsion angles; correlations between esds in cell parameters are only used when they are defined by crystal symmetry. An approximate (isotropic) treatment of cell esds is used for estimating esds involving l.s. planes.
Refinement. X-ray diffraction was recorded by Rigaku Oxford diffractometer with graphite-monochromated Mo *K*α radiation (0.71073 Å). *CrysAlis PRO* software (Agilent, 2014) was employed for data reduction. The structures were solved through intrinsic phasing and direct methods, employing *SHELXS* (Sheldrick, 2008) and *SHELXT* (Sheldrick, 2015a). Non-H atoms were refined anisotropically, while H atoms were geometrically placed and refined with isotropic displacement parameters using the riding model in the *SHELXL2019* program (Sheldrick, 2015*b*). Molecular graphics: *OLEX2* (Dolomanov *et al.*, 2009). The CIF file containing complete information on the studied structure has been deposited with CCDC under deposition numbers 2293931 and 2293932, and is freely available upon request *via* the following website: www.ccdc.cam.ac.uk/data_request/cif.

### (S)-(+)-1-(4-Bromophenyl)-N-[(4-methoxyphenyl)methylidene]ethylamine (I) Fractional atomic coordinates and isotropic or equivalent isotropic displacement parameters (Å^2^)

**Table:**

	*x*	*y*	*z*	*U*_iso_*/*U*_eq_	
Br1	0.2389 (2)	1.09813 (9)	0.52206 (2)	0.1427 (6)	
O1	0.3517 (9)	0.4597 (6)	0.82998 (17)	0.0963 (15)	
N1	0.0282 (11)	0.4666 (6)	0.65137 (19)	0.0844 (16)	
C1	0.0099 (12)	0.4345 (8)	0.6097 (2)	0.084 (2)	
H1	0.126103	0.348253	0.602570	0.101*	
C2	−0.2363 (13)	0.3682 (8)	0.6005 (2)	0.110 (2)	
H2A	−0.275397	0.278804	0.618246	0.165*	
H2B	−0.239708	0.326065	0.574324	0.165*	
H2C	−0.349134	0.457944	0.603169	0.165*	
C3	0.0642 (11)	0.5963 (8)	0.5870 (2)	0.0720 (17)	
C4	0.2300 (13)	0.6028 (7)	0.55835 (18)	0.0832 (17)	
H4	0.309999	0.504562	0.551551	0.100*	
C5	0.2826 (15)	0.7505 (9)	0.53913 (19)	0.0932 (19)	
H5	0.398557	0.751716	0.519944	0.112*	
C6	0.1649 (13)	0.8944 (7)	0.5483 (2)	0.084 (2)	
C7	−0.0019 (15)	0.8945 (10)	0.5770 (3)	0.112 (3)	
H7	−0.084855	0.992153	0.583272	0.135*	
C8	−0.0434 (15)	0.7452 (9)	0.5965 (2)	0.113 (3)	
H8	−0.149839	0.745846	0.617120	0.136*	
C9	0.1914 (11)	0.3938 (7)	0.6701 (2)	0.0779 (18)	
H9	0.292991	0.323549	0.656296	0.093*	
C10	0.2308 (11)	0.4124 (6)	0.71138 (19)	0.0671 (15)	
C11	0.4256 (12)	0.3396 (8)	0.7274 (3)	0.083 (2)	
H11	0.529551	0.280840	0.711440	0.100*	
C12	0.4736 (12)	0.3504 (8)	0.7669 (3)	0.083 (2)	
H12	0.607953	0.299154	0.777088	0.099*	
C13	0.3233 (12)	0.4364 (8)	0.7909 (2)	0.0732 (17)	
C14	0.1213 (12)	0.5094 (8)	0.7750 (2)	0.080 (2)	
H14	0.015617	0.565927	0.791160	0.096*	
C15	0.0768 (11)	0.4989 (7)	0.7360 (2)	0.0722 (19)	
H15	−0.057410	0.549886	0.725702	0.087*	
C16	0.5553 (14)	0.3902 (13)	0.8476 (3)	0.137 (3)	
H16A	0.549264	0.409011	0.875188	0.206*	
H16B	0.693720	0.443191	0.837058	0.206*	
H16C	0.561135	0.271122	0.842523	0.206*	

### (S)-(+)-1-(4-Bromophenyl)-N-[(4-methoxyphenyl)methylidene]ethylamine (I) Atomic displacement parameters (Å^2^)

**Table:**

	*U*^11^	*U*^22^	*U*^33^	*U*^12^	*U*^13^	*U*^23^
Br1	0.2380 (12)	0.0887 (5)	0.1013 (7)	−0.0090 (8)	0.0088 (9)	0.0093 (4)
O1	0.098 (4)	0.097 (3)	0.094 (4)	−0.004 (3)	0.010 (3)	0.018 (3)
N1	0.091 (4)	0.079 (3)	0.084 (5)	0.011 (3)	0.020 (4)	−0.002 (3)
C1	0.084 (5)	0.070 (4)	0.099 (6)	0.018 (4)	0.016 (4)	−0.001 (4)
C2	0.101 (5)	0.115 (5)	0.114 (6)	−0.007 (6)	0.002 (6)	−0.007 (4)
C3	0.069 (4)	0.070 (4)	0.077 (5)	0.008 (4)	0.004 (4)	−0.010 (4)
C4	0.091 (4)	0.081 (4)	0.078 (4)	0.020 (5)	0.007 (5)	−0.004 (3)
C5	0.109 (5)	0.095 (5)	0.075 (4)	0.002 (5)	0.022 (5)	−0.005 (4)
C6	0.115 (6)	0.065 (4)	0.072 (5)	0.001 (4)	−0.013 (4)	−0.003 (3)
C7	0.135 (7)	0.081 (5)	0.121 (7)	0.038 (5)	0.036 (6)	−0.003 (5)
C8	0.119 (6)	0.089 (5)	0.132 (7)	0.025 (5)	0.047 (6)	0.007 (5)
C9	0.070 (4)	0.056 (3)	0.108 (6)	0.012 (4)	0.016 (4)	0.004 (3)
C10	0.060 (4)	0.047 (3)	0.094 (5)	0.006 (4)	0.025 (4)	0.007 (3)
C11	0.075 (5)	0.064 (4)	0.111 (7)	0.022 (3)	0.020 (5)	0.007 (4)
C12	0.066 (4)	0.066 (4)	0.117 (7)	0.012 (4)	0.009 (5)	0.020 (4)
C13	0.070 (4)	0.056 (4)	0.094 (6)	−0.005 (3)	0.010 (4)	0.016 (4)
C14	0.077 (5)	0.062 (4)	0.102 (7)	0.000 (4)	0.030 (5)	0.010 (4)
C15	0.057 (4)	0.060 (4)	0.099 (6)	0.001 (3)	0.013 (4)	0.012 (4)
C16	0.104 (6)	0.190 (9)	0.117 (8)	−0.001 (7)	0.002 (6)	0.047 (7)

### (S)-(+)-1-(4-Bromophenyl)-N-[(4-methoxyphenyl)methylidene]ethylamine (I) Geometric parameters (Å, º)

**Table:**

Br1—C6	1.895 (6)	C7—H7	0.9300
O1—C13	1.363 (8)	C7—C8	1.379 (10)
O1—C16	1.413 (8)	C8—H8	0.9300
N1—C1	1.456 (8)	C9—H9	0.9300
N1—C9	1.265 (7)	C9—C10	1.444 (8)
C1—H1	0.9800	C10—C11	1.361 (9)
C1—C2	1.523 (9)	C10—C15	1.393 (8)
C1—C3	1.532 (9)	C11—H11	0.9300
C2—H2A	0.9600	C11—C12	1.387 (9)
C2—H2B	0.9600	C12—H12	0.9300
C2—H2C	0.9600	C12—C13	1.367 (9)
C3—C4	1.362 (8)	C13—C14	1.393 (8)
C3—C8	1.367 (8)	C14—H14	0.9300
C4—H4	0.9300	C14—C15	1.369 (8)
C4—C5	1.377 (8)	C15—H15	0.9300
C5—H5	0.9300	C16—H16A	0.9600
C5—C6	1.357 (9)	C16—H16B	0.9600
C6—C7	1.367 (9)	C16—H16C	0.9600
			
C13—O1—C16	117.7 (6)	C3—C8—H8	118.3
C9—N1—C1	118.1 (6)	C7—C8—H8	118.3
N1—C1—H1	108.7	N1—C9—H9	117.8
N1—C1—C2	109.2 (6)	N1—C9—C10	124.5 (6)
N1—C1—C3	109.9 (5)	C10—C9—H9	117.8
C2—C1—H1	108.7	C11—C10—C9	118.7 (6)
C2—C1—C3	111.5 (6)	C11—C10—C15	118.0 (7)
C3—C1—H1	108.7	C15—C10—C9	123.3 (6)
C1—C2—H2A	109.5	C10—C11—H11	119.0
C1—C2—H2B	109.5	C10—C11—C12	121.9 (6)
C1—C2—H2C	109.5	C12—C11—H11	119.0
H2A—C2—H2B	109.5	C11—C12—H12	120.0
H2A—C2—H2C	109.5	C13—C12—C11	120.0 (7)
H2B—C2—H2C	109.5	C13—C12—H12	120.0
C4—C3—C1	122.5 (6)	O1—C13—C12	126.0 (7)
C4—C3—C8	116.5 (6)	O1—C13—C14	115.2 (7)
C8—C3—C1	120.8 (6)	C12—C13—C14	118.8 (8)
C3—C4—H4	119.1	C13—C14—H14	119.7
C3—C4—C5	121.9 (6)	C15—C14—C13	120.7 (7)
C5—C4—H4	119.1	C15—C14—H14	119.7
C4—C5—H5	120.1	C10—C15—H15	119.7
C6—C5—C4	119.8 (7)	C14—C15—C10	120.6 (6)
C6—C5—H5	120.1	C14—C15—H15	119.7
C5—C6—Br1	119.8 (6)	O1—C16—H16A	109.5
C5—C6—C7	120.5 (6)	O1—C16—H16B	109.5
C7—C6—Br1	119.7 (6)	O1—C16—H16C	109.5
C6—C7—H7	121.1	H16A—C16—H16B	109.5
C6—C7—C8	117.9 (7)	H16A—C16—H16C	109.5
C8—C7—H7	121.1	H16B—C16—H16C	109.5
C3—C8—C7	123.3 (7)		
			
Br1—C6—C7—C8	−177.3 (6)	C6—C7—C8—C3	−3.7 (13)
O1—C13—C14—C15	178.1 (6)	C8—C3—C4—C5	−1.6 (10)
N1—C1—C3—C4	126.6 (7)	C9—N1—C1—C2	123.7 (6)
N1—C1—C3—C8	−49.3 (9)	C9—N1—C1—C3	−113.7 (6)
N1—C9—C10—C11	−174.0 (6)	C9—C10—C11—C12	−179.2 (6)
N1—C9—C10—C15	7.2 (9)	C9—C10—C15—C14	178.7 (5)
C1—N1—C9—C10	−179.6 (6)	C10—C11—C12—C13	−0.1 (10)
C1—C3—C4—C5	−177.7 (7)	C11—C10—C15—C14	−0.1 (8)
C1—C3—C8—C7	−179.8 (8)	C11—C12—C13—O1	−178.6 (6)
C2—C1—C3—C4	−112.2 (7)	C11—C12—C13—C14	1.1 (9)
C2—C1—C3—C8	72.0 (9)	C12—C13—C14—C15	−1.6 (8)
C3—C4—C5—C6	−1.1 (11)	C13—C14—C15—C10	1.1 (9)
C4—C3—C8—C7	4.1 (12)	C15—C10—C11—C12	−0.4 (9)
C4—C5—C6—Br1	179.6 (5)	C16—O1—C13—C12	0.5 (9)
C4—C5—C6—C7	1.5 (11)	C16—O1—C13—C14	−179.2 (6)
C5—C6—C7—C8	0.8 (12)		

### Dichloridobis{(S)-(+)-1-(4-bromophenyl)-N-[(4-methoxyphenyl)methylidene]ethylamine-κN}palladium(II) (II) Crystal data

**Table:**

[PdCl_2_(C_16_H_16_BrNO)_2_]	*F*(000) = 1616
*M**_r_* = 813.71	*D*_x_ = 1.677 Mg m^−^^3^
Monoclinic, *P*2_1_	Mo *K*α radiation, λ = 0.71073 Å
*a* = 9.0493 (4) Å	Cell parameters from 5092 reflections
*b* = 25.1365 (8) Å	θ = 1.6–25.9°
*c* = 14.1725 (7) Å	µ = 3.25 mm^−^^1^
β = 90.185 (4)°	*T* = 293 K
*V* = 3223.8 (2) Å^3^	Prism, clear orange
*Z* = 4	0.58 × 0.14 × 0.11 mm

### Dichloridobis{(S)-(+)-1-(4-bromophenyl)-N-[(4-methoxyphenyl)methylidene]ethylamine-κN}palladium(II) (II) Data collection

**Table:**

Rigaku Xcalibur Atlas Gemini diffractometer	8864 reflections with *I* > 2σ(*I*)
Detector resolution: 10.5564 pixels mm^-1^	*R*_int_ = 0.047
ω scans	θ_max_ = 26.4°, θ_min_ = 1.6°
Absorption correction: analytical (CrysAlis PRO; Rigaku OD, 2015)	*h* = −11→11
*T*_min_ = 0.406, *T*_max_ = 0.755	*k* = −31→31
21980 measured reflections	*l* = −17→17
12072 independent reflections	

### Dichloridobis{(S)-(+)-1-(4-bromophenyl)-N-[(4-methoxyphenyl)methylidene]ethylamine-κN}palladium(II) (II) Refinement

**Table:**

Refinement on *F*^2^	Hydrogen site location: inferred from neighbouring sites
Least-squares matrix: full	H-atom parameters constrained
*R*[*F*^2^ > 2σ(*F*^2^)] = 0.047	*w* = 1/[σ^2^(*F*_o_^2^) + (0.0407*P*)^2^] where *P* = (*F*_o_^2^ + 2*F*_c_^2^)/3
*wR*(*F*^2^) = 0.107	(Δ/σ)_max_ < 0.001
*S* = 1.02	Δρ_max_ = 0.78 e Å^−^^3^
12072 reflections	Δρ_min_ = −0.64 e Å^−^^3^
767 parameters	Absolute structure: Flack *x* determined using 2945 quotients [(I+)-(I-)]/[(I+)+(I-)] (Parsons *et al.*, 2013)
41 restraints	Absolute structure parameter: 0.005 (7)

### Dichloridobis{(S)-(+)-1-(4-bromophenyl)-N-[(4-methoxyphenyl)methylidene]ethylamine-κN}palladium(II) (II) Special details

**Table:**

Geometry. All esds (except the esd in the dihedral angle between two l.s. planes) are estimated using the full covariance matrix. The cell esds are taken into account individually in the estimation of esds in distances, angles and torsion angles; correlations between esds in cell parameters are only used when they are defined by crystal symmetry. An approximate (isotropic) treatment of cell esds is used for estimating esds involving l.s. planes.

### Dichloridobis{(S)-(+)-1-(4-bromophenyl)-N-[(4-methoxyphenyl)methylidene]ethylamine-κN}palladium(II) (II) Fractional atomic coordinates and isotropic or equivalent isotropic displacement parameters (Å^2^)

**Table:**

	*x*	*y*	*z*	*U*_iso_*/*U*_eq_	Occ. (<1)
Pd1	0.21529 (6)	0.74835 (4)	0.54298 (4)	0.04229 (16)	
Br1	0.09895 (17)	0.47281 (6)	0.41034 (13)	0.1115 (5)	
Br2	0.19435 (18)	0.97812 (7)	0.95910 (13)	0.1193 (6)	
Cl1	0.4129 (2)	0.79352 (12)	0.60901 (19)	0.0622 (7)	
Cl2	0.0102 (3)	0.70667 (11)	0.48069 (19)	0.0629 (7)	
O1	−0.0318 (10)	0.9452 (3)	0.2847 (6)	0.093 (3)	
O2	0.4183 (10)	0.5627 (3)	0.8521 (6)	0.094 (3)	
N1	0.3261 (7)	0.7338 (3)	0.4206 (5)	0.0471 (19)	
N2	0.0996 (7)	0.7663 (3)	0.6598 (5)	0.0427 (17)	
C1	0.4276 (9)	0.6881 (4)	0.4111 (6)	0.050 (2)	
H1	0.470334	0.690204	0.347706	0.060*	
C2	0.5557 (10)	0.6938 (5)	0.4812 (8)	0.068 (3)	
H2A	0.606080	0.726801	0.470159	0.103*	
H2B	0.623367	0.664767	0.473054	0.103*	
H2C	0.517813	0.693354	0.544407	0.103*	
C3	0.3457 (11)	0.6355 (4)	0.4169 (7)	0.054 (2)	
C4	0.3561 (12)	0.6007 (4)	0.4896 (8)	0.063 (3)	
H4	0.415864	0.609578	0.540705	0.075*	
C5	0.2824 (13)	0.5528 (5)	0.4914 (9)	0.073 (3)	
H5	0.289488	0.530240	0.543269	0.088*	
C6	0.1984 (12)	0.5394 (5)	0.4147 (9)	0.072 (3)	
C7	0.1832 (14)	0.5725 (5)	0.3393 (9)	0.081 (4)	
H7	0.124321	0.563028	0.288140	0.098*	
C8	0.2588 (13)	0.6214 (5)	0.3407 (8)	0.072 (3)	
H8	0.250107	0.644461	0.289687	0.087*	
C9	0.2962 (10)	0.7597 (4)	0.3434 (7)	0.059 (3)	
H9	0.335659	0.745504	0.288318	0.070*	
C10	0.2097 (11)	0.8076 (4)	0.3320 (7)	0.051 (2)	
C11	0.1418 (11)	0.8157 (4)	0.2428 (7)	0.059 (3)	
H11	0.152394	0.790139	0.195824	0.071*	
C12	0.0614 (12)	0.8605 (5)	0.2249 (8)	0.071 (3)	
H12	0.016891	0.865327	0.166251	0.085*	
C13	0.0465 (12)	0.8986 (5)	0.2946 (9)	0.069 (3)	
C14	0.1144 (13)	0.8916 (5)	0.3833 (8)	0.071 (3)	
H14	0.104445	0.917200	0.430218	0.086*	
C15	0.1945 (11)	0.8469 (4)	0.3988 (7)	0.054 (2)	
H15	0.241141	0.842639	0.456846	0.065*	
C16	−0.1024 (16)	0.9544 (6)	0.1980 (11)	0.110 (5)	
H16A	−0.030042	0.955783	0.148680	0.165*	
H16B	−0.154563	0.987657	0.200558	0.165*	
H16C	−0.171069	0.926165	0.185370	0.165*	
C17	0.0079 (10)	0.8169 (4)	0.6530 (7)	0.058 (3)	
H17	0.034250	0.833641	0.592965	0.070*	
C18	−0.1537 (10)	0.8028 (5)	0.6457 (8)	0.074 (3)	
H18A	−0.169043	0.779769	0.592623	0.110*	
H18B	−0.210696	0.834724	0.637570	0.110*	
H18C	−0.184220	0.785068	0.702379	0.110*	
C19	0.0469 (10)	0.8564 (4)	0.7287 (7)	0.049 (2)	
C20	0.1672 (12)	0.8902 (4)	0.7152 (9)	0.069 (3)	
H20	0.220125	0.887756	0.659225	0.083*	
C21	0.2101 (12)	0.9268 (5)	0.7808 (11)	0.081 (4)	
H21	0.288662	0.949786	0.769438	0.097*	
C22	0.1322 (12)	0.9286 (4)	0.8657 (10)	0.070 (3)	
C23	0.0178 (12)	0.8958 (4)	0.8815 (8)	0.063 (3)	
H23	−0.032726	0.897471	0.938525	0.076*	
C24	−0.0250 (10)	0.8598 (4)	0.8136 (7)	0.053 (2)	
H24	−0.104233	0.837216	0.825549	0.064*	
C25	0.1001 (9)	0.7427 (4)	0.7395 (7)	0.053 (2)	
H25	0.044226	0.758511	0.786852	0.064*	
C26	0.1787 (10)	0.6935 (4)	0.7656 (7)	0.049 (2)	
C27	0.2082 (14)	0.6864 (4)	0.8601 (7)	0.070 (3)	
H27	0.174250	0.711656	0.902884	0.084*	
C28	0.2851 (15)	0.6439 (4)	0.8932 (8)	0.080 (4)	
H28	0.304717	0.640378	0.957325	0.096*	
C29	0.3335 (14)	0.6059 (4)	0.8303 (9)	0.071 (3)	
C30	0.2992 (12)	0.6120 (4)	0.7360 (8)	0.067 (3)	
H30	0.328008	0.585832	0.693520	0.081*	
C31	0.2246 (12)	0.6551 (4)	0.7034 (7)	0.063 (3)	
H31	0.204690	0.658603	0.639247	0.075*	
C32	0.5031 (17)	0.5654 (6)	0.9352 (11)	0.119 (6)	
H32A	0.572788	0.594058	0.930347	0.178*	
H32B	0.555243	0.532487	0.943898	0.178*	
H32C	0.439284	0.571471	0.988073	0.178*	
Pd2	0.36904 (7)	0.24913 (4)	0.94152 (4)	0.04789 (17)	
C38	0.3235 (14)	0.0471 (4)	0.6439 (9)	0.070 (3)	
Br3	0.364 (2)	−0.0085 (7)	0.5546 (13)	0.094 (3)	0.65 (7)
Br3A	0.399 (4)	−0.0128 (10)	0.581 (3)	0.091 (5)	0.35 (7)
C54	0.3172 (16)	0.4618 (6)	1.0946 (14)	0.099 (4)	
Br4	0.2073 (11)	0.5275 (4)	1.0914 (13)	0.134 (2)	0.69 (5)
Br4A	0.203 (2)	0.5183 (11)	1.130 (3)	0.132 (6)	0.31 (5)
Cl3	0.1586 (3)	0.29551 (13)	0.9790 (2)	0.0800 (9)	
Cl4	0.5751 (3)	0.20070 (11)	0.89587 (18)	0.0588 (6)	
O3	0.4512 (10)	0.4464 (3)	0.6263 (7)	0.093 (3)	
O4	0.0781 (9)	0.0768 (4)	1.2618 (6)	0.086 (2)	
N3	0.2690 (8)	0.2233 (3)	0.8213 (5)	0.0495 (19)	
N4	0.4625 (8)	0.2695 (3)	1.0676 (6)	0.058 (2)	
C33	0.1983 (11)	0.1700 (4)	0.8354 (7)	0.054 (2)	
H33	0.234599	0.157492	0.896734	0.065*	
C34	0.0316 (13)	0.1763 (5)	0.8474 (9)	0.090 (4)	
H34A	0.012194	0.202178	0.895683	0.134*	
H34B	−0.010751	0.142790	0.865200	0.134*	
H34C	−0.011382	0.187987	0.788979	0.134*	
C35	0.2435 (12)	0.1276 (4)	0.7657 (8)	0.057 (3)	
C36	0.3656 (14)	0.0969 (5)	0.7829 (9)	0.077 (4)	
H36	0.423600	0.103915	0.835754	0.093*	
C37	0.4041 (13)	0.0560 (5)	0.7240 (11)	0.083 (4)	
H37	0.484591	0.034502	0.738515	0.100*	
C39	0.2048 (14)	0.0783 (5)	0.6222 (8)	0.072 (3)	
H39	0.150985	0.072329	0.567200	0.086*	
C40	0.1650 (12)	0.1194 (4)	0.6836 (7)	0.059 (3)	
H40	0.085305	0.141248	0.668938	0.071*	
C41	0.2517 (9)	0.2467 (5)	0.7425 (6)	0.050 (2)	
H41	0.196859	0.228664	0.696975	0.060*	
C42	0.3082 (10)	0.2979 (4)	0.7165 (7)	0.051 (2)	
C43	0.2573 (14)	0.3213 (5)	0.6329 (8)	0.075 (3)	
H43	0.188026	0.303556	0.595889	0.090*	
C44	0.3088 (16)	0.3701 (5)	0.6051 (9)	0.090 (4)	
H44	0.272499	0.385059	0.549715	0.109*	
C45	0.4136 (13)	0.3978 (4)	0.6574 (9)	0.069 (3)	
C46	0.4674 (14)	0.3742 (5)	0.7344 (9)	0.073 (3)	
H46	0.540281	0.391532	0.769118	0.088*	
C47	0.4187 (12)	0.3247 (4)	0.7647 (7)	0.062 (3)	
H47	0.460799	0.309421	0.818160	0.074*	
C48	0.5476 (16)	0.4773 (5)	0.6831 (11)	0.108 (5)	
H48A	0.567372	0.510527	0.652120	0.161*	
H48B	0.638560	0.458402	0.692543	0.161*	
H48C	0.502028	0.484005	0.742990	0.161*	
C49	0.5645 (12)	0.3178 (5)	1.0708 (7)	0.068 (3)	
H49	0.619881	0.314689	1.130119	0.082*	
C50	0.6791 (12)	0.3173 (5)	0.9939 (9)	0.077 (3)	
H50A	0.732134	0.284266	0.995645	0.116*	
H50B	0.746732	0.346263	1.003245	0.116*	
H50C	0.631238	0.321110	0.933674	0.116*	
C51	0.4751 (13)	0.3688 (5)	1.0794 (9)	0.071 (3)	
C52	0.4375 (15)	0.3966 (5)	0.9995 (9)	0.081 (4)	
H52	0.467196	0.384442	0.940517	0.097*	
C53	0.3548 (15)	0.4430 (5)	1.0071 (11)	0.098 (4)	
H53	0.325218	0.461207	0.953137	0.118*	
C55	0.3551 (14)	0.4339 (6)	1.1724 (11)	0.092 (4)	
H55	0.327605	0.446187	1.231727	0.111*	
C56	0.4335 (13)	0.3877 (6)	1.1645 (9)	0.080 (4)	
H56	0.458719	0.368964	1.218778	0.096*	
C57	0.4383 (10)	0.2452 (5)	1.1466 (7)	0.062 (2)	
H57	0.492908	0.258046	1.197414	0.074*	
C58	0.3422 (11)	0.2022 (4)	1.1687 (7)	0.057 (3)	
C59	0.3483 (14)	0.1851 (5)	1.2587 (8)	0.079 (3)	
H59	0.415497	0.201436	1.299165	0.094*	
C60	0.2608 (14)	0.1445 (5)	1.2953 (8)	0.079 (4)	
H60	0.264955	0.135815	1.359010	0.095*	
C61	0.1697 (12)	0.1182 (5)	1.2361 (9)	0.067 (3)	
C62	0.1586 (14)	0.1357 (6)	1.1431 (9)	0.090 (4)	
H62	0.092217	0.119069	1.102435	0.108*	
C63	0.2427 (13)	0.1765 (5)	1.1105 (8)	0.082 (4)	
H63	0.233130	0.187232	1.047992	0.098*	
C64	0.0669 (15)	0.0636 (5)	1.3598 (9)	0.093 (4)	
H64A	−0.004106	0.035613	1.367701	0.139*	
H64B	0.035858	0.094382	1.394607	0.139*	
H64C	0.161470	0.051983	1.382718	0.139*	

### Dichloridobis{(S)-(+)-1-(4-bromophenyl)-N-[(4-methoxyphenyl)methylidene]ethylamine-κN}palladium(II) (II) Atomic displacement parameters (Å^2^)

**Table:**

	*U*^11^	*U*^22^	*U*^33^	*U*^12^	*U*^13^	*U*^23^
Pd1	0.0440 (3)	0.0451 (3)	0.0377 (3)	−0.0002 (4)	0.0003 (3)	−0.0001 (4)
Br1	0.1098 (10)	0.0669 (8)	0.1580 (16)	−0.0210 (8)	0.0133 (10)	−0.0088 (9)
Br2	0.1224 (11)	0.0931 (11)	0.1420 (15)	−0.0070 (10)	−0.0413 (10)	−0.0405 (11)
Cl1	0.0490 (12)	0.0727 (17)	0.0649 (17)	−0.0041 (13)	−0.0059 (12)	−0.0156 (14)
Cl2	0.0557 (13)	0.0712 (17)	0.0616 (16)	−0.0095 (13)	−0.0043 (12)	−0.0152 (14)
O1	0.118 (6)	0.071 (5)	0.088 (7)	0.017 (5)	−0.018 (5)	0.017 (5)
O2	0.139 (7)	0.071 (5)	0.070 (6)	0.027 (6)	−0.031 (5)	0.003 (5)
N1	0.041 (3)	0.051 (5)	0.049 (4)	−0.003 (3)	0.007 (3)	−0.006 (4)
N2	0.046 (4)	0.051 (4)	0.031 (4)	−0.001 (3)	0.008 (3)	0.002 (3)
C1	0.046 (5)	0.061 (6)	0.043 (5)	0.002 (5)	0.013 (4)	−0.007 (5)
C2	0.053 (5)	0.083 (8)	0.069 (7)	0.000 (6)	−0.002 (5)	−0.009 (6)
C3	0.061 (6)	0.050 (6)	0.051 (6)	0.006 (5)	−0.001 (5)	−0.008 (5)
C4	0.070 (7)	0.065 (7)	0.053 (7)	0.007 (6)	0.002 (5)	−0.006 (6)
C5	0.088 (8)	0.061 (7)	0.070 (8)	−0.005 (6)	0.016 (7)	0.010 (6)
C6	0.071 (7)	0.061 (7)	0.084 (9)	−0.003 (6)	0.009 (6)	0.000 (7)
C7	0.097 (9)	0.074 (8)	0.073 (9)	−0.006 (7)	−0.017 (7)	−0.011 (7)
C8	0.090 (8)	0.061 (7)	0.066 (8)	0.011 (7)	−0.022 (7)	0.007 (6)
C9	0.070 (6)	0.065 (8)	0.041 (5)	−0.017 (6)	−0.003 (4)	−0.006 (5)
C10	0.065 (6)	0.047 (5)	0.042 (5)	−0.007 (5)	0.006 (5)	0.003 (5)
C11	0.085 (7)	0.058 (6)	0.035 (6)	−0.009 (6)	−0.001 (5)	0.002 (5)
C12	0.084 (8)	0.063 (7)	0.066 (8)	−0.007 (6)	−0.022 (6)	0.018 (6)
C13	0.075 (7)	0.055 (7)	0.077 (9)	−0.003 (6)	0.000 (6)	0.015 (6)
C14	0.103 (9)	0.053 (6)	0.058 (7)	−0.007 (7)	−0.016 (7)	0.007 (6)
C15	0.065 (6)	0.055 (6)	0.042 (6)	−0.009 (5)	−0.005 (5)	−0.008 (5)
C16	0.107 (10)	0.100 (11)	0.123 (13)	0.018 (9)	−0.027 (9)	0.031 (10)
C17	0.061 (6)	0.058 (6)	0.055 (6)	0.010 (5)	0.009 (5)	0.019 (5)
C18	0.055 (6)	0.084 (8)	0.082 (8)	0.012 (6)	−0.016 (6)	−0.011 (7)
C19	0.046 (5)	0.040 (5)	0.062 (7)	0.011 (4)	0.003 (5)	0.005 (5)
C20	0.066 (7)	0.056 (7)	0.084 (9)	−0.005 (6)	0.023 (6)	0.001 (6)
C21	0.058 (7)	0.052 (7)	0.134 (13)	0.000 (6)	−0.001 (8)	0.000 (8)
C22	0.059 (6)	0.056 (7)	0.094 (10)	0.010 (6)	−0.008 (7)	−0.021 (6)
C23	0.068 (6)	0.065 (7)	0.058 (7)	−0.005 (6)	0.002 (5)	−0.008 (6)
C24	0.047 (5)	0.051 (6)	0.063 (7)	0.000 (5)	0.018 (5)	−0.003 (5)
C25	0.059 (5)	0.042 (5)	0.060 (6)	−0.001 (5)	0.007 (4)	0.002 (5)
C26	0.062 (5)	0.040 (5)	0.046 (6)	−0.002 (5)	0.008 (5)	−0.001 (5)
C27	0.124 (9)	0.049 (6)	0.038 (6)	0.007 (7)	−0.012 (6)	−0.005 (5)
C28	0.139 (11)	0.054 (7)	0.048 (7)	−0.010 (7)	−0.019 (7)	0.000 (6)
C29	0.103 (9)	0.040 (6)	0.068 (8)	−0.003 (6)	−0.020 (7)	0.011 (6)
C30	0.085 (8)	0.057 (6)	0.059 (7)	0.021 (6)	−0.002 (6)	0.002 (6)
C31	0.097 (8)	0.046 (6)	0.045 (6)	0.011 (6)	−0.003 (6)	0.002 (5)
C32	0.128 (12)	0.089 (10)	0.137 (14)	0.009 (10)	−0.068 (11)	0.021 (10)
Pd2	0.0542 (3)	0.0495 (4)	0.0399 (3)	−0.0004 (4)	−0.0015 (3)	−0.0035 (4)
C38	0.085 (7)	0.048 (5)	0.077 (8)	−0.022 (6)	0.036 (6)	−0.008 (5)
Br3	0.108 (5)	0.076 (4)	0.099 (5)	−0.023 (3)	0.032 (4)	−0.033 (3)
Br3A	0.099 (8)	0.064 (3)	0.110 (11)	−0.016 (5)	0.048 (7)	−0.017 (6)
C54	0.098 (9)	0.074 (7)	0.127 (11)	−0.005 (6)	0.007 (8)	−0.024 (8)
Br4	0.137 (3)	0.109 (3)	0.156 (6)	0.035 (3)	0.044 (4)	0.000 (4)
Br4A	0.138 (6)	0.094 (7)	0.163 (14)	−0.006 (6)	0.055 (8)	−0.052 (8)
Cl3	0.0710 (16)	0.086 (2)	0.083 (2)	0.0181 (16)	−0.0006 (15)	−0.0281 (18)
Cl4	0.0606 (14)	0.0630 (15)	0.0529 (15)	0.0079 (13)	−0.0024 (12)	−0.0008 (13)
O3	0.117 (7)	0.064 (5)	0.098 (7)	−0.010 (5)	0.005 (5)	0.028 (5)
O4	0.094 (6)	0.091 (6)	0.073 (6)	−0.019 (5)	0.008 (5)	0.012 (5)
N3	0.057 (4)	0.046 (4)	0.046 (5)	0.001 (4)	−0.006 (4)	−0.004 (4)
N4	0.055 (4)	0.063 (5)	0.054 (5)	−0.010 (4)	−0.010 (4)	−0.007 (4)
C33	0.067 (6)	0.055 (6)	0.040 (5)	−0.005 (5)	0.001 (5)	0.002 (5)
C34	0.098 (9)	0.079 (8)	0.092 (10)	−0.032 (8)	0.034 (8)	−0.032 (8)
C35	0.069 (6)	0.042 (5)	0.060 (7)	−0.014 (5)	−0.009 (5)	0.000 (5)
C36	0.085 (8)	0.053 (6)	0.093 (10)	−0.001 (7)	−0.018 (7)	−0.009 (7)
C37	0.060 (7)	0.052 (7)	0.137 (13)	−0.007 (6)	0.012 (8)	−0.012 (8)
C39	0.103 (9)	0.059 (7)	0.054 (7)	−0.016 (7)	−0.005 (6)	0.003 (6)
C40	0.072 (7)	0.062 (6)	0.043 (6)	0.003 (6)	−0.014 (5)	0.004 (5)
C41	0.054 (4)	0.049 (5)	0.047 (5)	0.006 (6)	−0.009 (4)	−0.004 (6)
C42	0.062 (6)	0.047 (5)	0.044 (6)	0.011 (5)	0.000 (5)	0.005 (5)
C43	0.100 (9)	0.075 (8)	0.050 (7)	−0.003 (7)	−0.005 (6)	0.011 (6)
C44	0.129 (11)	0.074 (8)	0.068 (8)	−0.011 (8)	−0.030 (8)	0.038 (7)
C45	0.078 (7)	0.050 (6)	0.079 (9)	0.003 (6)	0.010 (6)	0.015 (6)
C46	0.094 (8)	0.058 (7)	0.068 (8)	−0.016 (6)	0.006 (7)	0.000 (6)
C47	0.080 (7)	0.057 (6)	0.048 (6)	−0.007 (6)	−0.006 (5)	0.005 (5)
C48	0.127 (11)	0.054 (7)	0.142 (14)	−0.014 (8)	0.026 (10)	0.001 (9)
C49	0.075 (7)	0.080 (8)	0.049 (6)	−0.028 (6)	−0.006 (6)	−0.002 (6)
C50	0.065 (6)	0.074 (8)	0.093 (9)	−0.013 (6)	−0.012 (6)	−0.006 (7)
C51	0.078 (7)	0.065 (7)	0.071 (9)	−0.021 (6)	0.003 (7)	−0.021 (7)
C52	0.125 (10)	0.067 (7)	0.052 (7)	0.000 (8)	0.005 (7)	−0.017 (6)
C53	0.111 (10)	0.074 (9)	0.109 (12)	−0.007 (8)	−0.015 (9)	−0.038 (9)
C55	0.077 (8)	0.096 (11)	0.104 (12)	−0.032 (8)	0.040 (8)	−0.030 (10)
C56	0.076 (8)	0.098 (10)	0.065 (8)	−0.032 (8)	0.001 (6)	−0.014 (8)
C57	0.070 (6)	0.068 (6)	0.047 (5)	0.007 (7)	−0.016 (5)	−0.010 (7)
C58	0.057 (5)	0.063 (6)	0.052 (6)	−0.001 (5)	−0.008 (5)	0.002 (5)
C59	0.104 (9)	0.080 (8)	0.052 (7)	−0.017 (8)	−0.018 (7)	0.009 (7)
C60	0.117 (10)	0.079 (8)	0.040 (6)	−0.010 (8)	−0.006 (7)	0.016 (6)
C61	0.065 (7)	0.071 (7)	0.066 (8)	0.003 (6)	−0.006 (6)	0.003 (6)
C62	0.090 (9)	0.122 (12)	0.057 (8)	−0.031 (9)	−0.009 (7)	−0.010 (8)
C63	0.098 (9)	0.104 (10)	0.044 (6)	−0.032 (8)	−0.008 (6)	−0.002 (7)
C64	0.114 (10)	0.084 (9)	0.080 (9)	−0.001 (8)	−0.006 (8)	0.018 (8)

### Dichloridobis{(S)-(+)-1-(4-bromophenyl)-N-[(4-methoxyphenyl)methylidene]ethylamine-κN}palladium(II) (II) Geometric parameters (Å, º)

**Table:**

Pd1—Cl1	2.313 (2)	Pd2—Cl4	2.321 (3)
Pd1—Cl2	2.305 (2)	Pd2—N3	2.034 (7)
Pd1—N1	2.040 (7)	Pd2—N4	2.040 (8)
Pd1—N2	2.013 (7)	C38—Br3	1.921 (17)
Br1—C6	1.902 (12)	C38—Br3A	1.88 (3)
Br2—C22	1.900 (11)	C38—C37	1.367 (17)
O1—C13	1.376 (13)	C38—C39	1.364 (16)
O1—C16	1.403 (14)	C54—Br4	1.929 (17)
O2—C29	1.365 (13)	C54—Br4A	1.83 (2)
O2—C32	1.405 (14)	C54—C53	1.37 (2)
N1—C1	1.477 (11)	C54—C55	1.35 (2)
N1—C9	1.301 (11)	O3—C45	1.342 (13)
N2—C17	1.521 (11)	O3—C48	1.417 (15)
N2—C25	1.276 (11)	O4—C61	1.380 (14)
C1—H1	0.9800	O4—C64	1.431 (14)
C1—C2	1.531 (12)	N3—C33	1.498 (12)
C1—C3	1.519 (13)	N3—C41	1.271 (11)
C2—H2A	0.9600	N4—C49	1.526 (12)
C2—H2B	0.9600	N4—C57	1.294 (12)
C2—H2C	0.9600	C33—H33	0.9800
C3—C4	1.355 (14)	C33—C34	1.527 (14)
C3—C8	1.380 (14)	C33—C35	1.510 (14)
C4—H4	0.9300	C34—H34A	0.9600
C4—C5	1.377 (15)	C34—H34B	0.9600
C5—H5	0.9300	C34—H34C	0.9600
C5—C6	1.367 (16)	C35—C36	1.370 (15)
C6—C7	1.360 (16)	C35—C40	1.376 (13)
C7—H7	0.9300	C36—H36	0.9300
C7—C8	1.406 (16)	C36—C37	1.369 (16)
C8—H8	0.9300	C37—H37	0.9300
C9—H9	0.9300	C39—H39	0.9300
C9—C10	1.445 (13)	C39—C40	1.399 (15)
C10—C11	1.419 (13)	C40—H40	0.9300
C10—C15	1.376 (13)	C41—H41	0.9300
C11—H11	0.9300	C41—C42	1.434 (14)
C11—C12	1.364 (14)	C42—C43	1.399 (13)
C12—H12	0.9300	C42—C47	1.385 (13)
C12—C13	1.383 (16)	C43—H43	0.9300
C13—C14	1.408 (15)	C43—C44	1.370 (16)
C14—H14	0.9300	C44—H44	0.9300
C14—C15	1.355 (14)	C44—C45	1.389 (17)
C15—H15	0.9300	C45—C46	1.333 (16)
C16—H16A	0.9600	C46—H46	0.9300
C16—H16B	0.9600	C46—C47	1.388 (15)
C16—H16C	0.9600	C47—H47	0.9300
C17—H17	0.9800	C48—H48A	0.9600
C17—C18	1.508 (13)	C48—H48B	0.9600
C17—C19	1.502 (14)	C48—H48C	0.9600
C18—H18A	0.9600	C49—H49	0.9800
C18—H18B	0.9600	C49—C50	1.507 (15)
C18—H18C	0.9600	C49—C51	1.520 (16)
C19—C20	1.396 (14)	C50—H50A	0.9600
C19—C24	1.372 (13)	C50—H50B	0.9600
C20—H20	0.9300	C50—H50C	0.9600
C20—C21	1.363 (17)	C51—C52	1.374 (16)
C21—H21	0.9300	C51—C56	1.352 (16)
C21—C22	1.398 (17)	C52—H52	0.9300
C22—C23	1.343 (15)	C52—C53	1.390 (17)
C23—H23	0.9300	C53—H53	0.9300
C23—C24	1.377 (14)	C55—H55	0.9300
C24—H24	0.9300	C55—C56	1.364 (18)
C25—H25	0.9300	C56—H56	0.9300
C25—C26	1.473 (13)	C57—H57	0.9300
C26—C27	1.376 (13)	C57—C58	1.424 (15)
C26—C31	1.374 (13)	C58—C59	1.348 (14)
C27—H27	0.9300	C58—C63	1.380 (14)
C27—C28	1.359 (15)	C59—H59	0.9300
C28—H28	0.9300	C59—C60	1.392 (16)
C28—C29	1.378 (16)	C60—H60	0.9300
C29—C30	1.380 (15)	C60—C61	1.348 (15)
C30—H30	0.9300	C61—C62	1.392 (16)
C30—C31	1.356 (13)	C62—H62	0.9300
C31—H31	0.9300	C62—C63	1.359 (16)
C32—H32A	0.9600	C63—H63	0.9300
C32—H32B	0.9600	C64—H64A	0.9600
C32—H32C	0.9600	C64—H64B	0.9600
Pd2—Cl3	2.297 (3)	C64—H64C	0.9600
			
Cl2—Pd1—Cl1	176.95 (10)	N3—Pd2—Cl4	87.4 (2)
N1—Pd1—Cl1	92.88 (19)	N3—Pd2—N4	175.2 (3)
N1—Pd1—Cl2	89.5 (2)	N4—Pd2—Cl3	90.6 (2)
N2—Pd1—Cl1	87.7 (2)	N4—Pd2—Cl4	92.6 (2)
N2—Pd1—Cl2	89.8 (2)	C37—C38—Br3	124.4 (13)
N2—Pd1—N1	176.5 (3)	C37—C38—Br3A	109.4 (19)
C13—O1—C16	117.6 (11)	C39—C38—Br3	114.9 (13)
C29—O2—C32	117.2 (10)	C39—C38—Br3A	129.9 (19)
C1—N1—Pd1	121.7 (6)	C39—C38—C37	120.7 (11)
C9—N1—Pd1	121.6 (7)	C53—C54—Br4	113.8 (15)
C9—N1—C1	116.1 (8)	C53—C54—Br4A	131 (2)
C17—N2—Pd1	114.9 (6)	C55—C54—Br4	126.4 (15)
C25—N2—Pd1	128.6 (6)	C55—C54—Br4A	109 (2)
C25—N2—C17	116.4 (8)	C55—C54—C53	119.8 (14)
N1—C1—H1	106.9	C45—O3—C48	118.0 (10)
N1—C1—C2	109.8 (8)	C61—O4—C64	118.4 (10)
N1—C1—C3	111.6 (7)	C33—N3—Pd2	111.3 (6)
C2—C1—H1	106.9	C41—N3—Pd2	129.9 (7)
C3—C1—H1	106.9	C41—N3—C33	118.6 (8)
C3—C1—C2	114.5 (9)	C49—N4—Pd2	118.4 (6)
C1—C2—H2A	109.5	C57—N4—Pd2	124.6 (7)
C1—C2—H2B	109.5	C57—N4—C49	117.0 (8)
C1—C2—H2C	109.5	N3—C33—H33	105.3
H2A—C2—H2B	109.5	N3—C33—C34	110.1 (9)
H2A—C2—H2C	109.5	N3—C33—C35	115.3 (8)
H2B—C2—H2C	109.5	C34—C33—H33	105.3
C4—C3—C1	124.8 (9)	C35—C33—H33	105.3
C4—C3—C8	117.8 (10)	C35—C33—C34	114.6 (9)
C8—C3—C1	117.3 (10)	C33—C34—H34A	109.5
C3—C4—H4	118.4	C33—C34—H34B	109.5
C3—C4—C5	123.1 (11)	C33—C34—H34C	109.5
C5—C4—H4	118.4	H34A—C34—H34B	109.5
C4—C5—H5	121.0	H34A—C34—H34C	109.5
C6—C5—C4	117.9 (11)	H34B—C34—H34C	109.5
C6—C5—H5	121.0	C36—C35—C33	120.1 (10)
C5—C6—Br1	120.3 (10)	C36—C35—C40	118.7 (11)
C7—C6—Br1	117.8 (10)	C40—C35—C33	121.3 (10)
C7—C6—C5	121.9 (11)	C35—C36—H36	119.3
C6—C7—H7	120.8	C37—C36—C35	121.4 (12)
C6—C7—C8	118.4 (11)	C37—C36—H36	119.3
C8—C7—H7	120.8	C38—C37—C36	119.6 (12)
C3—C8—C7	120.7 (11)	C38—C37—H37	120.2
C3—C8—H8	119.6	C36—C37—H37	120.2
C7—C8—H8	119.6	C38—C39—H39	120.4
N1—C9—H9	115.8	C38—C39—C40	119.2 (11)
N1—C9—C10	128.4 (9)	C40—C39—H39	120.4
C10—C9—H9	115.8	C35—C40—C39	120.3 (10)
C11—C10—C9	116.9 (9)	C35—C40—H40	119.9
C15—C10—C9	125.3 (9)	C39—C40—H40	119.9
C15—C10—C11	117.7 (9)	N3—C41—H41	116.6
C10—C11—H11	119.5	N3—C41—C42	126.7 (9)
C12—C11—C10	120.9 (10)	C42—C41—H41	116.6
C12—C11—H11	119.5	C43—C42—C41	118.6 (9)
C11—C12—H12	120.3	C47—C42—C41	124.6 (9)
C11—C12—C13	119.5 (10)	C47—C42—C43	116.7 (10)
C13—C12—H12	120.3	C42—C43—H43	119.7
O1—C13—C12	124.7 (11)	C44—C43—C42	120.5 (12)
O1—C13—C14	114.7 (11)	C44—C43—H43	119.7
C12—C13—C14	120.6 (11)	C43—C44—H44	119.1
C13—C14—H14	120.7	C43—C44—C45	121.9 (11)
C15—C14—C13	118.6 (11)	C45—C44—H44	119.1
C15—C14—H14	120.7	O3—C45—C44	117.0 (11)
C10—C15—H15	118.7	C46—C45—O3	125.6 (12)
C14—C15—C10	122.6 (9)	C46—C45—C44	117.4 (11)
C14—C15—H15	118.7	C45—C46—H46	118.8
O1—C16—H16A	109.5	C45—C46—C47	122.5 (12)
O1—C16—H16B	109.5	C47—C46—H46	118.8
O1—C16—H16C	109.5	C42—C47—C46	120.8 (10)
H16A—C16—H16B	109.5	C42—C47—H47	119.6
H16A—C16—H16C	109.5	C46—C47—H47	119.6
H16B—C16—H16C	109.5	O3—C48—H48A	109.5
N2—C17—H17	106.2	O3—C48—H48B	109.5
C18—C17—N2	109.7 (8)	O3—C48—H48C	109.5
C18—C17—H17	106.2	H48A—C48—H48B	109.5
C19—C17—N2	112.4 (7)	H48A—C48—H48C	109.5
C19—C17—H17	106.2	H48B—C48—H48C	109.5
C19—C17—C18	115.4 (9)	N4—C49—H49	105.6
C17—C18—H18A	109.5	C50—C49—N4	112.9 (9)
C17—C18—H18B	109.5	C50—C49—H49	105.6
C17—C18—H18C	109.5	C50—C49—C51	115.6 (10)
H18A—C18—H18B	109.5	C51—C49—N4	110.5 (8)
H18A—C18—H18C	109.5	C51—C49—H49	105.6
H18B—C18—H18C	109.5	C49—C50—H50A	109.5
C20—C19—C17	119.1 (10)	C49—C50—H50B	109.5
C24—C19—C17	123.8 (9)	C49—C50—H50C	109.5
C24—C19—C20	117.0 (10)	H50A—C50—H50B	109.5
C19—C20—H20	118.7	H50A—C50—H50C	109.5
C21—C20—C19	122.6 (12)	H50B—C50—H50C	109.5
C21—C20—H20	118.7	C52—C51—C49	119.5 (11)
C20—C21—H21	121.1	C56—C51—C49	121.3 (13)
C20—C21—C22	117.8 (11)	C56—C51—C52	119.2 (13)
C22—C21—H21	121.1	C51—C52—H52	120.2
C21—C22—Br2	118.2 (9)	C51—C52—C53	119.7 (13)
C23—C22—Br2	120.8 (10)	C53—C52—H52	120.2
C23—C22—C21	121.0 (11)	C54—C53—C52	119.6 (15)
C22—C23—H23	119.9	C54—C53—H53	120.2
C22—C23—C24	120.2 (11)	C52—C53—H53	120.2
C24—C23—H23	119.9	C54—C55—H55	119.8
C19—C24—C23	121.4 (10)	C54—C55—C56	120.3 (15)
C19—C24—H24	119.3	C56—C55—H55	119.8
C23—C24—H24	119.3	C51—C56—C55	121.3 (14)
N2—C25—H25	116.1	C51—C56—H56	119.3
N2—C25—C26	127.8 (9)	C55—C56—H56	119.3
C26—C25—H25	116.1	N4—C57—H57	114.6
C27—C26—C25	116.4 (9)	N4—C57—C58	130.9 (9)
C31—C26—C25	125.2 (9)	C58—C57—H57	114.6
C31—C26—C27	118.4 (9)	C59—C58—C57	115.3 (9)
C26—C27—H27	118.8	C59—C58—C63	116.2 (11)
C28—C27—C26	122.3 (10)	C63—C58—C57	128.5 (10)
C28—C27—H27	118.8	C58—C59—H59	117.8
C27—C28—H28	120.5	C58—C59—C60	124.4 (11)
C27—C28—C29	119.1 (10)	C60—C59—H59	117.8
C29—C28—H28	120.5	C59—C60—H60	120.8
O2—C29—C28	125.7 (11)	C61—C60—C59	118.4 (11)
O2—C29—C30	115.7 (11)	C61—C60—H60	120.8
C28—C29—C30	118.6 (10)	O4—C61—C62	116.5 (11)
C29—C30—H30	119.0	C60—C61—O4	124.9 (11)
C31—C30—C29	122.0 (10)	C60—C61—C62	118.5 (12)
C31—C30—H30	119.0	C61—C62—H62	119.3
C26—C31—H31	120.2	C63—C62—C61	121.4 (11)
C30—C31—C26	119.6 (9)	C63—C62—H62	119.3
C30—C31—H31	120.2	C58—C63—H63	119.5
O2—C32—H32A	109.5	C62—C63—C58	121.0 (11)
O2—C32—H32B	109.5	C62—C63—H63	119.5
O2—C32—H32C	109.5	O4—C64—H64A	109.5
H32A—C32—H32B	109.5	O4—C64—H64B	109.5
H32A—C32—H32C	109.5	O4—C64—H64C	109.5
H32B—C32—H32C	109.5	H64A—C64—H64B	109.5
Cl3—Pd2—Cl4	176.77 (10)	H64A—C64—H64C	109.5
N3—Pd2—Cl3	89.4 (2)	H64B—C64—H64C	109.5
			
Pd1—N1—C1—C2	−62.3 (9)	Pd2—N3—C41—C42	3.9 (15)
Pd1—N1—C1—C3	65.7 (9)	Pd2—N4—C49—C50	−48.5 (11)
Pd1—N1—C9—C10	13.6 (13)	Pd2—N4—C49—C51	82.8 (10)
Pd1—N2—C17—C18	107.5 (8)	Pd2—N4—C57—C58	−3.2 (16)
Pd1—N2—C17—C19	−122.7 (7)	C38—C39—C40—C35	−1.0 (17)
Pd1—N2—C25—C26	−4.5 (14)	Br3—C38—C37—C36	−180.0 (10)
Br1—C6—C7—C8	−178.6 (9)	Br3—C38—C39—C40	179.0 (9)
Br2—C22—C23—C24	−179.3 (8)	Br3A—C38—C37—C36	−178.0 (12)
O1—C13—C14—C15	−179.6 (10)	Br3A—C38—C39—C40	176.4 (12)
O2—C29—C30—C31	−175.3 (11)	C54—C55—C56—C51	0.0 (19)
N1—C1—C3—C4	−110.2 (11)	Br4—C54—C53—C52	178.4 (11)
N1—C1—C3—C8	72.4 (11)	Br4—C54—C55—C56	−179.9 (10)
N1—C9—C10—C11	−154.2 (9)	Br4A—C54—C53—C52	−176.1 (14)
N1—C9—C10—C15	29.4 (16)	Br4A—C54—C55—C56	176.1 (11)
N2—C17—C19—C20	83.5 (11)	O3—C45—C46—C47	177.1 (11)
N2—C17—C19—C24	−93.4 (10)	O4—C61—C62—C63	179.4 (12)
N2—C25—C26—C27	156.6 (10)	N3—C33—C35—C36	87.7 (12)
N2—C25—C26—C31	−23.0 (16)	N3—C33—C35—C40	−91.4 (12)
C1—N1—C9—C10	−175.5 (8)	N3—C41—C42—C43	−169.2 (10)
C1—C3—C4—C5	−178.6 (10)	N3—C41—C42—C47	15.9 (16)
C1—C3—C8—C7	177.9 (10)	N4—C49—C51—C52	−94.2 (12)
C2—C1—C3—C4	15.2 (14)	N4—C49—C51—C56	86.8 (12)
C2—C1—C3—C8	−162.2 (9)	N4—C57—C58—C59	177.6 (12)
C3—C4—C5—C6	2.0 (17)	N4—C57—C58—C63	−3 (2)
C4—C3—C8—C7	0.3 (17)	C33—N3—C41—C42	178.8 (9)
C4—C5—C6—Br1	177.8 (8)	C33—C35—C36—C37	176.0 (11)
C4—C5—C6—C7	−1.9 (18)	C33—C35—C40—C39	−177.0 (10)
C5—C6—C7—C8	1.1 (19)	C34—C33—C35—C36	−142.9 (11)
C6—C7—C8—C3	−0.3 (19)	C34—C33—C35—C40	38.0 (15)
C8—C3—C4—C5	−1.2 (17)	C35—C36—C37—C38	3.1 (19)
C9—N1—C1—C2	126.8 (9)	C36—C35—C40—C39	3.8 (17)
C9—N1—C1—C3	−105.2 (9)	C37—C38—C39—C40	−0.7 (17)
C9—C10—C11—C12	−178.2 (9)	C39—C38—C37—C36	−0.3 (18)
C9—C10—C15—C14	178.5 (10)	C40—C35—C36—C37	−4.9 (18)
C10—C11—C12—C13	0.4 (16)	C41—N3—C33—C34	−73.5 (11)
C11—C10—C15—C14	2.1 (16)	C41—N3—C33—C35	58.0 (12)
C11—C12—C13—O1	−180.0 (10)	C41—C42—C43—C44	−179.7 (11)
C11—C12—C13—C14	0.4 (18)	C41—C42—C47—C46	179.7 (10)
C12—C13—C14—C15	0.0 (18)	C42—C43—C44—C45	1 (2)
C13—C14—C15—C10	−1.3 (17)	C43—C42—C47—C46	4.6 (16)
C15—C10—C11—C12	−1.6 (15)	C43—C44—C45—O3	−176.9 (13)
C16—O1—C13—C12	0.6 (17)	C43—C44—C45—C46	3 (2)
C16—O1—C13—C14	−179.8 (11)	C44—C45—C46—C47	−2 (2)
C17—N2—C25—C26	−179.9 (8)	C45—C46—C47—C42	−1.4 (19)
C17—C19—C20—C21	−179.5 (10)	C47—C42—C43—C44	−4.3 (18)
C17—C19—C24—C23	178.3 (9)	C48—O3—C45—C44	174.3 (11)
C18—C17—C19—C20	−149.7 (9)	C48—O3—C45—C46	−5.1 (19)
C18—C17—C19—C24	33.5 (13)	C49—N4—C57—C58	174.9 (10)
C19—C20—C21—C22	2.1 (17)	C49—C51—C52—C53	179.5 (11)
C20—C19—C24—C23	1.4 (14)	C49—C51—C56—C55	179.0 (10)
C20—C21—C22—Br2	178.3 (8)	C50—C49—C51—C52	35.7 (15)
C20—C21—C22—C23	−0.8 (17)	C50—C49—C51—C56	−143.4 (11)
C21—C22—C23—C24	−0.2 (17)	C51—C52—C53—C54	3 (2)
C22—C23—C24—C19	−0.2 (16)	C52—C51—C56—C55	−0.1 (17)
C24—C19—C20—C21	−2.5 (15)	C53—C54—C55—C56	1 (2)
C25—N2—C17—C18	−76.5 (11)	C55—C54—C53—C52	−3 (2)
C25—N2—C17—C19	53.3 (11)	C56—C51—C52—C53	−1.4 (18)
C25—C26—C27—C28	−177.3 (11)	C57—N4—C49—C50	133.4 (10)
C25—C26—C31—C30	178.5 (10)	C57—N4—C49—C51	−95.4 (11)
C26—C27—C28—C29	−1.1 (19)	C57—C58—C59—C60	178.2 (11)
C27—C26—C31—C30	−1.1 (16)	C57—C58—C63—C62	180.0 (12)
C27—C28—C29—O2	176.3 (11)	C58—C59—C60—C61	4 (2)
C27—C28—C29—C30	−1.4 (19)	C59—C58—C63—C62	−1.1 (19)
C28—C29—C30—C31	2.6 (19)	C59—C60—C61—O4	179.2 (11)
C29—C30—C31—C26	−1.3 (18)	C59—C60—C61—C62	−5.2 (19)
C31—C26—C27—C28	2.3 (17)	C60—C61—C62—C63	3 (2)
C32—O2—C29—C28	−22.3 (19)	C61—C62—C63—C58	0 (2)
C32—O2—C29—C30	155.4 (12)	C63—C58—C59—C60	−0.9 (19)
Pd2—N3—C33—C34	102.2 (8)	C64—O4—C61—C60	6.2 (17)
Pd2—N3—C33—C35	−126.2 (7)	C64—O4—C61—C62	−169.4 (12)

### Dichloridobis{(S)-(+)-1-(4-bromophenyl)-N-[(4-methoxyphenyl)methylidene]ethylamine-κN}palladium(II) (II) Hydrogen-bond geometry (Å, º)

**Table:**

*D*—H···*A*	*D*—H	H···*A*	*D*···*A*	*D*—H···*A*
C2—H2*C*···Cl1^i^	0.96	2.84	3.354 (12)	114
C7—H7···Br4^i^	0.93	2.61	3.27 (4)	129
C9—H9···Cl4^ii^	0.93	2.96	3.883	173
C18—H18···Cl2^iii^	0.96	2.92	3.679	137
C21—H21···Br3^iii^	0.93	3.01	3.64 (4)	127
C28—H28···Cl4^iv^	0.93	2.79	3.543 (12)	139
C32—H32*B*···Br2^v^	0.96	2.98	3.811	146
C32—H32*C*···Br4^v^	0.96	2.79	3.61 (2)	143
C34—H34*A*···Cl3^v^	0.96	2.94	3.709	138
C48—H48*A*···Br3^ii^	0.96	3.04	3.483	110
C48—H48···O2^v^	0.96	2.63	3.426	141
C50—H50*A*···Cl4^v^	0.96	2.90	3.376	112
C64—H64*A*···O1^vi^	0.96	2.57	3.284 (15)	131

Symmetry codes: (i) *x*, *y*, *z*−1; (ii) −*x*+1, *y*+1/2, −*z*+1; (iii) *x*, *y*+1, *z*; (iv) −*x*+1, *y*+1/2, −*z*+2; (v) −*x*+1, *y*−1/2, −*z*+2; (vi) *x*, *y*−1, *z*+1.
